# Aligning digital biomarker definitions in psychiatry with the National Institute of Mental Health Research Domain Criteria framework

**DOI:** 10.1038/s44277-024-00017-6

**Published:** 2024-10-13

**Authors:** Shai Mulinari

**Affiliations:** Department of Sociology, Lund University, Lund, Sweden

**Keywords:** Biomarkers, Scientific community

## Abstract

The field of biological psychiatry faces a growing influx of digital biomarkers spanning self-report, social, behavioral, cognitive, and physiological indicators of various mental health conditions. However, the definition of “digital biomarker,” particularly the “bio-” component, remains unclear. This article reviews the terminology of digital biomarkers in psychiatry and argues for the reservation of the term exclusively for measures of biological parameters with a plausible pathway connecting to the disease or condition of interest to enhance terminological clarity and consistency with conventional definitions of biomarker, short for biological marker. While the distinction between biological and non-biological parameters may blur at the edges, the Research Domain Criteria (RDoC) developed by the US National Institute of Mental Health offers a valuable heuristic. The RDoC distinguishes between biological (genes, molecules, cells, neural circuits, physiology) and non-biological (broadly understood behavior and self-report) units of analysis. Aligning digital biomarker definitions in psychiatry with the RDoC framework would mark a significant shift from the current broad usage, where almost any digitally measured characteristic, when used as an indicator, qualifies as a digital biomarker.

## Lay Summary

This work explores the growing use of digital tools to track mental health, such as apps that measure behavior or physiology. It points out that the term "digital biomarker" is often used too broadly and argues it should only refer to biological data that can be directly linked to a mental health condition. By using a clearer definition, the field can better focus on meaningful indicators that align with biological psychiatry standards.

## Introduction

In medicine, debates over terminology are commonplace, often focused on establishing shared definitions for concepts and standards. These definitions are crucial not only for research and medical practice but also for effective communication with other research fields, professions, funders, regulators, and patients.

**Fig. 1:**
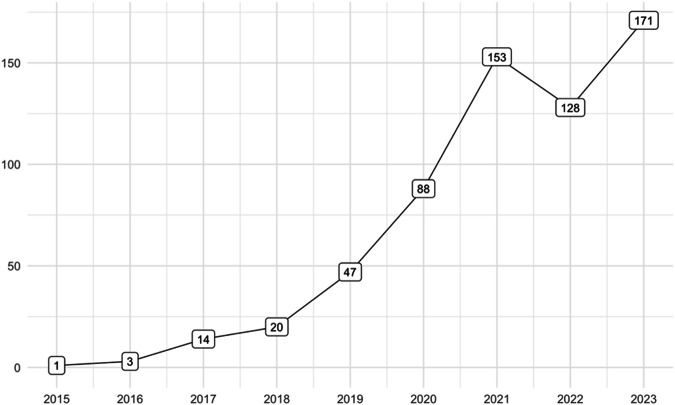
Articles in PubMed with ‘digital biomarker/s’ in title or abstract.

The definition of digital biomarkers is no exception [1]. Since its initial definition in 2015 as relevant digital signatures of pathological manifestations [2], the concept has been subject to the proposal of numerous definitions. A recent systematic mapping identified a staggering 127 definitions across 128 articles [3]. Meanwhile, the number of articles incorporating “digital biomarker” in their titles or abstracts has surged (Fig. 1), mirroring the rapid growth of the global digital biomarker market, projected to exceed US $20 billion by the next decade [4].

Remarkably, this growing research and commercial interest persists despite ongoing ambiguity about the term’s precise meaning [1]. The systematic mapping [3] pinpointed three primary areas of definitional divergence. First, the *type of data* collected, such as “objective, quantifiable, physiological, and behavioral data” or “all human data.” Second, the *data collection method*, such as “from everyday technologies including smartphones, wearable devices, social media, and computer interactions” or “by wearable, implantable, or ingestible devices and sensors.” Third, the *purpose of digital biomarkers*, such as “indicators of health outcomes” or to “gather more objective measures of social interaction and understanding than caregivers- or self-reports.”

In psychiatric digital biomarkers, an additional dimension comes to the forefront – the interpretation of “bio-” within the digital biomarker framework [5]. Biological psychiatrists and neuroscientists may be surprised that the defining feature of a digital biomarker is not that it is a biologically derived indicator, but rather its ability to be recorded and quantified using digital technologies. This expands the biomarker concept to include a widening array of human data, from genetic information to digitally recorded behaviors and self-reports [6].

This broadening of the biomarker concept beyond genuine biological parameters calls for a critical examination of digital biomarker terminology. The term digital biomarker fuses two powerful concepts from distinct domains: “digital” and “bio-.” In psychiatry, as in other medical fields, “digital” has become a source of hope and anticipation, potentially leading to uncritical acceptance of anything bearing this label [6]. Similarly, “biology” has long been associated with optimism in psychiatry, often viewed as offering deeper explanations for complex phenomena [7]. The combination of “digital” and “bio-” creates a particularly potent term, heightening the need for precision in its use.

Contrary to its current usage, this article advocates for a standard medical definition of digital biomarkers limited to digital measures of biological parameters plausibly linked to the disease or condition of interest. While the distinction between biological and non-biological parameters may blur at the edges, the Research Domain Criteria (RDoC) framework developed by the US National Institute of Mental Health (NIMH) provides a valuable heuristic [8]. Significantly, many proposed psychiatric digital biomarkers derive from measures of broadly understood behavior and self-report, which do not classify as biological units of analysis within the RDoC framework.

## From conventional to unconventional definitions of biomarkers

Medical dictionaries typically define biomarkers, short for *biological markers*, as measurements of biological parameters used to indicate disease-related outcomes or processes (Table 1).

**Table 1:** Definitions of biomarker in common medical dictionaries.

Medical dictionary	Definition of biomarker
The Oxford Concise Medical Dictionary (10 ed.)	A normal metabolite that, when present in abnormal concentrations in certain body fluids, can indicate the presence of a particular disease or toxicological condition. For example, abnormal concentrations of glucose in the blood can be indicative of diabetes mellitus.
Dorland’s Illustrated Medical Dictionary (33rd ed.)	A biological molecule used as a marker for the substance or process of interest.
Online Harvard Medical Dictionary of Health Terms	A distinctive biological indicator of an event, process, or condition.
Black’s Medical Dictionary (43 ed.)	A material measurable in blood or other body fluids which indicates the presence or absence of a disease or condition. Biomarkers are used in diagnosis, more commonly in surveillance of someone being treated or observed for a known condition.
Merriam-Webster’s Medical Dictionary (new ed.)	A biological or biologically derived indicator (as a metabolite in the body) of a process, event or condition (as disease or exposure to a toxin)
A Dictionary of Biomedicine (2nd ed.)	1. Any biological feature that provides information about the status of a system, either an organism or a whole ecosystem. Biomarkers for the concentration of heavy metals in soil as pollution indicators are an example.
	2. A more restricted usage is for molecules (e.g. proteins, antigens) that may indicate disease if found at increased levels in the blood.

Conventional definitions of biomarkers in psychiatry are similar. For instance, Singh and Rose [7] define biomarkers as “biological means of predicting not only the development of a disorder but also its course and outcome.” Examples in psychiatry include physiological markers like skin conductivity and brain activity, genetic markers, and endophenotypes—intermediate traits in the causal chain between genes and diseases, such as abnormal eye-tracking movements in patients with schizophrenia.

However, as Teixeira et al. [9] summarize, identifying biomarkers in psychiatry has proven elusive despite advances in neuroscience. A primary obstacle to progress is attributed to the absence of an objective diagnostic and classification system to address the heterogeneity of psychiatric disorders [8]. Unlike other medical disciplines, which typically categorize diseases by biological abnormalities, psychiatry relies on subjective behavioral tests and self-reporting. This approach groups patients with highly variable symptom profiles—for example, there are approximately 1,500 symptom combinations leading to a Major Depressive Disorder diagnosis [10]. Consequently, patient cohorts are too heterogeneously large [8], diluting any strong biological “signals”. This dilemma has been termed psychiatry’s Catch-22—biomarkers are essential to develop valid diagnostic categories, but to identify biomarkers, valid diagnostic categories are needed [11]. Currently, psychiatry lacks both, leading to an “unbreakable chain of circularity” [12].

The NIMH developed the RDoC as an effort to break this circularity, seeking to offer an alternative to the prevailing diagnostic classification systems [8]. Recently, researchers, including former NIMH Director Thomas Insel, who participated in developing the RDoC, proposed that novel digital technologies, such as smartphones, offer another avenue for breaking this deadlock by enabling unprecedented data collection and analysis of behavior, cognition, and emotions [13]. Yet, at the same time, the very definition of biomarkers appears to be changing with the introduction of novel digital technologies [6], confounding the RDoC framework.

To exemplify this, consider the landmark paper by Torous et al. [14], which suggested that digital data collection would span the seven nested units of analysis outlined in the RDoC framework [8], namely “physiology, behavior, and self-report,” as well as, in the near future, also across “biological units at the level of genes, molecules, cells, and neural circuits.” It would appear logical, and in keeping with the RDoC framework, to refer only to measurements of the latter biological units as unequivocal biomarkers. However, in a subsequent article, Torous et al. [15] proposed that smartphone-based data collection and analysis of users’ survey responses, call logs, GPS, screen use, and heart rate would result in distinct “self-report,” “social,” “behavioral,” “cognitive,” and “physiological” digital biomarkers. Most of these measures are not inherently biological, with the possible exception of physiological measures.

## The example of smartphone user data as digital biomarkers

The perspective presented by Torous et al. [15] on digital biomarkers is now widespread. Psychiatric digital biomarker researchers routinely seek non-biological metrics capable of predicting individuals’ mental health. In a groundbreaking study, Jacobson et al. [16]. demonstrated how smartphone sensor data might help forecast social anxiety symptom severity. The authors utilized incoming and outgoing calls and text messages, as well as accelerometer data, which were categorized as distinct “call,” “text message,” and “accelerometer” biomarkers. For example, “call biomarkers” were computed from five data streams: (1) total number of calls; (2) percentage of missed calls; (3) percentage of calls when the phone was idle (indicating interruption of current tasks); (4) the number of total persons contacted by the participants, and (5) the time differences between calls (indicating call return times) [16].

In a related example, Choudhary et al. [17] analyzed smartphone app usage “to create a novel digital biomarker detecting Generalized Anxiety Disorder.” This digital biomarker analysis categorized users’ smartphone apps into 12 types, such as “passive information consumption apps,” “active messaging and communications apps,” “games,” “navigation utilities,” and “health and fitness-related apps.” Data calculations included the daily frequency and duration of app use by type, said to provide insights into users’ time spent on digital activities like shopping, gaming, online dating, and communication.

Measures of smartphone users’ geolocation is another common non-biological source for digital biomarkers. For example, studies have used GPS metrics such as median daily distance traveled, time spent away from home, and median and maximum distance traveled from home, to derive digital biomarkers of schizophrenia [18] and posttraumatic stress disorder [19]. More broadly, a systematic review of the literature of geolocation-derived digital biomarkers of bipolar disorder and schizophrenia reported biomarkers of *mobility* (e.g. distance traveled), *location (*e.g. locations visited), *regularity* (e.g. location routine patterns), and *activities* (e.g. employment, shopping, sports, socializing, and recreation) [20].

## Confusion about the meaning of “Bio-” in digital biomarker

These examples show that digital biomarkers, as currently understood, encompass measurements that extend beyond biological parameters. This raises a fundamental question: if “bio-” does not refer to the inherent biological property of the marker, what does it denote? One interpretation suggests it refers to the *outcome*, yet most psychiatric outcomes are not biological either [13]. This has led to two main perspectives on the necessary biological properties of outcome variables [21]:

“Some authors argue that digital biomarkers should be directly linked to biological variables such as those related to genetics, epigenetics, endocrinology, immunology, etc., while others defend a broader definition of digital (bio)markers that encompass human behaviors, psychological states, and environmental factors that influence biology to varying degrees.”

It is noteworthy that Bodenstein et al. [21] refer to the latter category as “digital (bio)markers,” indicating a degree of ambivalence. Their main reference is the article by Montag et al. [5], which represents a significant effort to clarify the meaning of “bio-” in psychiatric digital biomarkers. Montag et al. [5] propose a distinction between direct and indirect digital biomarkers: direct biomarkers predict biological variables, such as neural circuits, while indirect biomarkers predict non-biological variables that may be indirectly linked to biological systems. They further clarify their view by noting that many psychiatric outcomes rely on behavioral and psychological assessments that do not yield genuine biological variables. Therefore, digital footprints predicting typical psychiatric outcomes serve as examples of indirect biomarkers.

This definition prompts reflection, as it reverses the traditional understanding of biomarkers [6]. It implies that a biomarker is defined not by a biological variable predicting a relevant medical outcome, but by digitized behavior predicting a biological variable. This shift in meaning disqualifies much of psychiatric digital biomarker research as “true” biomarker research, along with the broader body of non-digital biomarker research. Yet, this stance contradicts the view of the RDoC designers, who cautioned that “biomarker search might be stymied by a false assumption of a ‘gold standard’ biological event” as the outcome to predict [8].

## Towards a better definition of digital biomarker in psychiatry

What drives this shift in understanding of biomarkers? While broader epistemological and technological changes contribute [6], one culprit is the ambiguity in the definition of biomarker in the BEST Resource Glossary published in 2016. Originally presented by FDA and NIH leaders as “a dynamic document [that] will foster a shared understanding among all who study and use biomarkers and clinical assessments” [22], this glossary has fallen short of this goal. It defines biomarkers as:

“A defined characteristic that is measured as an indicator of normal biological processes, pathogenic processes, or biological responses to an exposure or intervention, including therapeutic interventions. Biomarkers may include molecular, histologic, radiographic, or physiologic characteristics. A biomarker is not a measure of how an individual feels, functions, or survives.”

This definition’s first part is often cited or alluded to by researchers [3], regulators [1], and industry [23] when defining digital biomarkers. However, it is often misinterpreted to suggest that any “defined characteristic” measured with digital technologies qualifies as a potential digital biomarker. The glossary, however, also emphasizes the need for *biologic plausibility*—the presence of biological, physiological, or pathological pathways plausibly explaining correlations. Further, the definition builds on the FDA-NIH Biomarker Working Group’s seminal publication from 2001: “Biomarkers and Surrogate Endpoints” [24], which frames biomarkers as “biological measurements” obtained from analytical tools “to assess biological parameters”, and which explicitly defines biomarkers as *biological markers*:

“Biological marker (biomarker): A characteristic that is objectively measured and evaluated as an indicator of normal biological processes, pathogenic processes, or pharmacologic responses to a therapeutic intervention.”

To avoid confusion, the BEST Resource Glossary should be updated to clearly distinguish between biological markers (biomarkers) and non-biological markers. This clarification would also mean that digital biomarkers must represent biological markers, not just any digitally collected marker.

Incorporating the RDoC’s nested units of analysis would further clarify the meaning of “bio-” in psychiatric digital biomarkers. While early research may tolerate loose terminology, ambiguous use of “bio-” risks diluting scientific language and reducing precision [23, 24]. Misuse of “bio-” can also hinder interdisciplinary research, causing confusion between fields like biology, medicine, computer science, psychology, and sociology [6].

There are also societal risks with ambiguous use of “bio-”. Public misunderstandings and false perceptions of scientific claims may arise [6], as seen, for example, with the marketing of pseudo-scientific products such as “bio-cosmetics” or “bio-identical hormones” [25]. Misuse can also reinforce deterministic narratives, oversimplifying complex behaviors by attributing them solely to biology [7]. Historically, invocations of “biology” have lent unwarranted authority to claims supporting social inequalities, such as racial hierarchies [26]. Additionally, labeling non-biological data, such as digital behaviors, as “bio-” could allow companies or states to collect personal data under the guise of biological monitoring [27].

Precision and scientific rigor in the use of “bio-” are crucial to mitigating these risks. Authors, reviewers, and journal editors in digital psychiatry can all play a vital role in ensuring accurate and consistent use of digital biomarker terminology in research and public discourse.
